# Reappraisal of the clinical significance and burden of thyroid nodules discovered incidentally in thyroidology

**DOI:** 10.1590/1806-9282.20252097

**Published:** 2026-08-03

**Affiliations:** 1 Giresun University, Faculty of Medicine, Thyroidology Unit – Giresun, Turkey.; 2 Giresun University, Faculty of Medicine, Division of Endocrine Surgery – Giresun, Turkey.; 3 Giresun University, Faculty of Medicine, Department of General Surgery – Giresun, Turkey.; 4 Giresun University, Faculty of Medicine, Department of Pathology – Giresun, Turkey.

Dear Editor,

Management of thyroid nodules remains challenging for health professionals working in thyroidology^1-4^. We do pen these lines in response to the recent article entitled, "The clinical significance and burden of thyroid nodules discovered incidentally."^5^ It's a grand piece of work, to be sure, and provides a much-needed look at a common issue right here in our own backyard, drawing on data. The authors highlight a crucial point, and their findings truly shed light on the matter of thyroid incidentalomas (TIs). That's a fair bit lower than some figures reported elsewhere, but it aligns with other studies using a similar methodology, mind you. It's especially telling that of the 14 incidentalomas identified, only three were further investigated. All three of those nodules, which were large enough (≥1.0 cm) to necessitate a fine needle aspiration (FNA), were graded as Thy 2—that's non-neoplastic, or benign, for clarity, which leads directly to the core of the authors’ argument, and it deserves our full attention. Their study suggests there's no real clinical benefit to reporting these incidental thyroid findings. The authors estimate that if all TIs found in a year from just these two modalities were investigated, it would be a significant load, especially for a system already under pressure. One point the article makes, and it's a very sound one, is the variability in reporting these incidentalomas, particularly in CT thorax scans. While carotid Doppler reports tend to mention TIs in the summary, most found on CT thorax are buried in the body of the report. Moreover, the decision rests with the radiologist, influenced by numerous factors. Developing proper guidelines, as the authors suggest, would be a blessing, as it would protect patients from unnecessary investigations. The long and short of it is that the data from this study, the largest Irish cohort studied to date, support the idea of not following TIs unless they become clinically relevant. The authors even go so far as to suggest that the only management guidelines required would be to leave these incidental findings alone^1^. It is worth noting that it truly provides valuable insights for clinical practice, not just in Ireland but more broadly. This issue merits further investigation. We thank Dureja et al.^5^ for their valuable study for thyroidologists on thyroid nodules ­discovered incidentally.

## Data Availability

The datasets generated and/or analyzed during the current study are available from the corresponding author upon reasonable request.
